# Nutritional intake and determinants of nutritional quality changes from pregnancy to postpartum—a longitudinal study

**DOI:** 10.1002/fsn3.3838

**Published:** 2023-11-20

**Authors:** Mathilda Forsby, Erik Hulander, Anna Amberntsson, Petra Brembeck, Anna Winkvist, Linnea Bärebring, Hanna Augustin

**Affiliations:** ^1^ Institute of Medicine, University of Gothenburg Gothenburg Sweden; ^2^ Institute of Health and Care Sciences, University of Gothenburg Gothenburg Sweden

**Keywords:** breastfeeding, diet, dietary reference intake, lactation, maternal health, Nordic countries, nutrients

## Abstract

Nutrient requirements vary across the reproductive cycle, but research on changes in nutritional intake and quality from pregnancy to beyond the lactation period is limited. Thus, we aimed to study nutritional intake and quality changes, among Swedish pregnant participants from late pregnancy to 18 months postpartum and to study the determinants of nutritional quality changes. Participants (*n* = 72) were studied longitudinally from the third trimester of pregnancy and postpartum (2 weeks 4, 12, and 18 months postpartum). At each visit, participant characteristics and 4‐day food diaries were collected. Nutritional quality was assessed by energy adjusted Nutrient Rich Food Index 11.3. Linear mixed models were used to analyze the determinants of change in nutritional quality. Intakes of carbohydrate energy percentage (E%), fiber, vitamin A, vitamin C, and potassium were higher in the third trimester compared to postpartum, whereas intakes of E% protein and monounsaturated fat were lower. Adherence to recommended intakes was low at all study visits for saturated fat (4%–11%), fiber (15%–39%), vitamin D (8%–14%), folate (0%–2%), and iron (6%–21%). Overall, nutritional quality did not differ significantly from third trimester to postpartum. Shorter duration (<4 months) of lactation was negatively related to nutritional quality changes, whereas higher age was positively related to changes. In conclusion, nutritional intake from pregnancy to postpartum changed, whereas quality remained relatively stable, with age and lactation duration as determinants. Identification of people at risk of adverse dietary changes from pregnancy to the postpartum period should be further addressed in future larger and more diverse study populations.

## Graphical abstract

The aims of this study were to investigate changes in nutritional intake and quality, from late pregnancy to 18 months postpartum among Swedish pregnant participants, and to study the determinants of nutritional quality changes. Nutritional quality was assessed by energy adjusted Nutrient Rich Food Index 11.3. Less than one‐fifth of the participants reached the recommended intake of vitamin D, folate, and iron, and lactation duration was a main determinant for changes in nutritional quality.

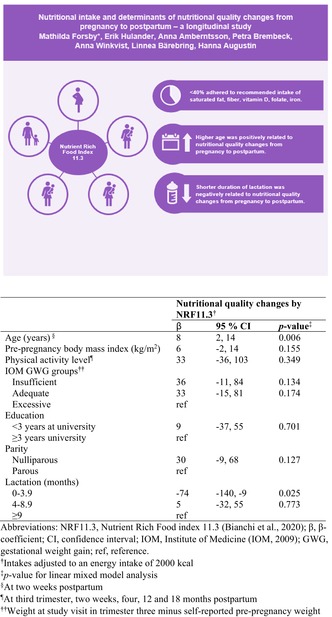

## 1 INTRODUCTION

A healthy diet and maintaining adequate nutritional status during pregnancy are essential for the health of both pregnant individuals and their children. Energy and micronutrient requirements increase during pregnancy to support fetal growth and development (Barker, 1997; Kaiser & Allen, 2008; Konde, 2020). Moreover, a varied and nutrient‐rich diet after delivery is important to meet the increased energy expenditure during lactation and to ensure sufficient nutritional intake for the infant (Konde, 2020). In Sweden, dietary advice for the general population, as well as for pregnant and lactating individuals are formulated by the Swedish National Food Agency (Konde, 2020), based on the Nordic Nutrition Recommendations (NNR) (Nordic Council of Ministers, 2014). Routine visits within the Swedish antenatal care system involve health‐promoting consultations about dietary habits and assessments of nutritional status. These routine visits are a valuable resource for implementing nutritional guidelines (Swedish Society of Obstetrics and Gynecology, 2016).

Poor nutritional quality during pregnancy has been associated with adverse outcomes, including preeclampsia (Brantsaeter et al., 2009), gestational diabetes (Amati et al., 2019), and birth complications (Chia et al., 2019). We have previously shown that high intake of caloric beverages, snacks, fish, and bread, as well as poor fat quality and high intake of unhealthy foods (sweets, cakes, soft drinks, and french fries) during pregnancy were associated with excessive gestational weight gain (GWG) (Augustin et al., 2020; Bärebring, Brembeck, et al., 2016). Furthermore, poor dietary quality during pregnancy has been reported in the north of Sweden and even poorer dietary quality after delivery (Wennberg et al., 2016). The poorer dietary quality was mainly explained by decreased intakes of fruits and vegetables and increased intakes of sweets, cakes, cookies, crisps, and ice cream from pregnancy to postpartum (Wennberg et al., 2016). Also, results from the Norwegian Mother, Father, and Child Cohort demonstrated that a higher nutritional quality during pregnancy was related to lower odds of the baby being born small for gestational age (Hillesund et al., 2014). Others have found that a higher maternal dietary quality during pregnancy and lactation has been associated with lower weight‐for‐length Z‐scores at birth and up to 6 months of age, and reduced infant adiposity of 6 months age, important factors for later growth and body composition development (Tahir et al., 2019).

Longitudinal studies on nutritional intake and nutritional quality from pregnancy to postpartum in Sweden are lacking. Findings from the Swedish birth cohort NICE (Nutritional impact on immunological maturation during childhood in relation to the environment) showed that more than half of the females reported inadequate intakes of vitamin D, iron, folate, selenium, and iodine during pregnancy (Stråvik et al., 2019). Adequate intake of these nutrients can possibly reduce the risk of several pregnancy complications with potential long‐term health consequences during both pregnancy and childhood (Allen, 2000; Bärebring, Bullarbo, et al., 2016; Bärebring, Bullarbo, et al., 2018; Modzelewska et al., 2021; Tamura & Picciano, 2006). A German study showed that dietary quality, assessed by Nutritional Health Score, was lower 3 months postpartum than during pregnancy (Poulain et al., 2021). However, no significant changes in dietary quality were seen between pregnancy and 12 months postpartum. Determinants of nutritional quality changes from pregnancy to postpartum have, so far, rarely been studied. Overall, studies have suggested that younger age and lower socioeconomic status are associated with poorer dietary quality during pregnancy (Doyle et al., 2017) and postpartum (Poulain et al., 2021). Furthermore, higher pre‐pregnancy BMI has been associated with increased dietary quality between three and 12 months postpartum (Poulain et al., 2021).

Previous studies on dietary intake during pregnancy and lactation have focused on specific nutrients or indices mainly based on certain foods or food groups (Arvizu et al., 2019; Augustin et al., 2020; Bärebring, Brembeck, et al., 2016; Grundt et al., 2017; Lee et al., 2018; Massari et al., 2020; Roth et al., 2018; Stråvik et al., 2019; Von Ruesten et al., 2014; Wennberg et al., 2016). However, studies assessing dietary quality involving multiple nutrients are less common. An index based on nutrient quality is easier to compare internationally since nutrients are generic, in contrast to a food‐based index where consumed foods can differ between cultures. The Nutrient Rich Food index (NRF) covers a wide range of nutrients and has been validated against the Healthy Eating Index (Fulgoni 3rd et al., 2009). Recently, the NRF index was applied to assess diet quality during pregnancy in Japan (Imai et al., 2021). A Swedish version of the NRF index (NRF 11.3) has also been developed and applied in a Swedish population‐based cohort, but not among pregnant individuals (Strid et al., 2021). NRF 11.3 proved to rank foods most coherently with the Swedish dietary guidelines compared to other variants of the NRF index (Bianchi et al., 2020). The Swedish studies assessing NRF indices emphasized the importance of aligning the selection of nutrients within the NRF index with population‐specific characteristics to ensure an accurate representation of country‐specific guidelines (Bianchi et al., 2020; Strid et al., 2021).

Nutrient requirements vary throughout the reproductive cycle (Konde, 2020). Studies investigating longitudinal changes in nutritional intake and quality during this critical period remain relatively scarce (Crozier et al., 2009; Lebrun et al., 2019; Poulain et al., 2021; Wennberg et al., 2016). Hence, the aims of this study were to (1) study nutritional intake and quality changes from the third trimester of pregnancy to 18 months postpartum among Swedish individuals, and (2) explore the determinants of nutritional quality changes during this period. We hypothesized that there would be changes in nutritional quality from pregnancy to the postpartum period, driven by factors associated with nutrient intake and quality across the reproductive cycle (Augustin et al., 2020; Northstone et al., 2008; Poulain et al., 2021; Stråvik et al., 2019; Wiltheiss et al., 2013).

## 2 METHODS

### 2.1 Study population and data collection

Data were collected in the prospective cohort study BUGA (Benmetabolism Under Graviditet och Amning: Bone metabolism During Pregnancy and Lactation) where 95 pregnant participants were recruited between July 2008 and July 2011 (Brembeck et al., 2013). Recruitment was carried out through advertisement on a web page directed to pregnant individuals and through posters at antenatal care clinics as well as other public locations, e.g., libraries in Gothenburg, Sweden. Inclusion criteria in the BUGA study were healthy individuals, aged 24–40 years, in gestational week 35–37 who were not using any prescribed drugs known to affect bone mineral metabolism (Brembeck et al., 2013). Individuals reporting recent bone fractures, pre‐eclampsia, gestational diabetes, multifetal pregnancy, pregnancy during the last 18 months before start of the present pregnancy, or breastfeeding during the last 12 months before entering the present pregnancy were excluded. Verbal and written study information were provided, and written informed consent was obtained from all participants. The study was conducted according to the guidelines in the Declaration of Helsinki. The Regional Ethics Committee in Gothenburg approved all procedures (protocol number 129‐08).

The first study visit took place in gestational week 35–37 at the Department of Internal Medicine and Clinical Nutrition at the University of Gothenburg. Thereafter the participants visited the department at 2 weeks‐, 4 months‐, 12 months‐ and 18 months postpartum. At each visit, height (standardized wall stadiometer) and weight (Tanita, BWB‐800MA, Rex Fredriksbergs Vaegtfabrik) were measured after an overnight fast. Additionally, information on medical history, use of medication or dietary supplements, age, education, parity, physical activity level (PAL), estimated pre‐pregnancy weight, and information on current lactation habits was collected via self‐administered questionnaires. PAL was estimated by a validated 10‐graded scale, where 1 corresponded to PAL 1.3 and 10 to PAL 2.2 (Lagerros et al., 2012). GWG was calculated as the measured third trimester weight minus the self‐reported pre‐pregnancy weight. At each study visit, the participants were instructed to complete a four‐day food diary.

### 2.2 Nutritional intake

The participants were instructed to complete the food diary during four consecutive days (at least one non‐working day) and were encouraged not to change their food habits during food recording. They were instructed to quantify all consumed meals, including drinks and food items, either using kitchen scale, household measures or photographic templates of portion size provided by the Swedish portion guide “Matmallen” (The National Food Agency, 1997). The participants were not asked to specify amounts of added salt or spices. Trained study personnel controlled the food diaries after completion and in case of any ambiguities, the participant was contacted for clarification.

**FIGURE 1:**
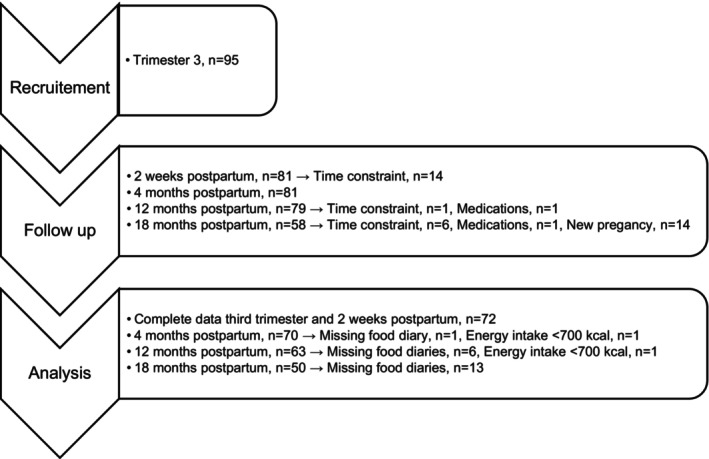
Study population including reasons for dropout, missing data and excluded cases.

Nutrient intake was calculated using the software Dietist XP version 3.1 (Kost och näringsdata, Bromma, Sweden, version 2009‐11‐10). The intake of added sugar was calculated using data from the National Food Agency (Wanselius et al., 2019). Reported average daily intake was compared to Recommended intake (RI) during pregnancy and lactation, and for female individuals of reproductive age, and average requirement (AR) for female individuals of reproductive age, derived from NNR (Nordic Council of Ministers, 2014). Participants who had not completed the food diaries in the third trimester of pregnancy and at 2 weeks postpartum were excluded from the analyses. Food diaries with mean estimated energy intake <700 kcal were excluded from the analyses (Figure 1).

### 2.3 Nutritional quality

The NRF11.3 index (Bianchi et al., 2020) was used to estimate nutritional quality from the four‐day food diary at each study visit (Figure S1). In total, the index included 14 nutrients: 11 nutrients to encourage (protein, fiber, vitamin A, C, D, E, folate, calcium, iron, magnesium, and potassium) and three nutrients to limit (saturated fat, added sugar, and sodium). The encouraged nutrients, labeled as NR11, contributed to a positive score, while the nutrients to limit, labeled as LIM3, contributed to a negative score. NR11 and LIM3 were calculated as the estimated average intake over the four consecutive days. Dietary reference intake (DRI) and maximum recommended intake (MRI) for each nutrient were derived from NNR 2012 (Nordic Council of Ministers, 2014) as the RI for female individuals of reproductive age. Capping was used to avoid that an excessive intake of one nutrient would compensate for deficiency of another. All encouraged nutrients were capped when the intakes exceeded 100% of DRI, with the exception for fiber due to no specified upper limit in NNR 2012 (Nordic Council of Ministers, 2014). Fifteen percent of energy from proteins (E%) was set as the reference value (Strid et al., 2021) as it is the mean of the recommended interval 10–20 E%. The score of NRF11.3 was calculated as the sum of the ratios between NR11 and DRI subtracted by the sum of the ratios between LIM3 and MRI. A higher score indicates a better adherence to the nutritional recommendations in NNR (Nordic Council of Ministers, 2014). Provided that the intake of NR11 reaches DRI and the intake of LIM3 do not exceed MRI, the score of NRF11.3 is 1100. The score may slightly exceed 1100, as capping was not used for fiber intake. As the intention was to examine nutritional intake and quality from food and beverages, the intake of dietary supplements was not included in the NRF11.3 index analyses. The NRF11.3 index was calculated using relative intakes adjusted to an energy intake of 2000 kcal (referred to as nutritional quality).

### 2.4 Determinants of changes in nutritional quality

Potential determinants of changes in nutritional quality were selected based on findings in previous studies on nutritional quality, demographic and lifestyle factors throughout the reproduction cycle (Augustin et al., 2020; Northstone et al., 2008; Poulain et al., 2021; Stråvik et al., 2019; Wiltheiss et al., 2013). Age, pre‐pregnancy BMI, PAL at each study visit, education, GWG, parity and lactation were identified and selected as potential determinants. Age, pre‐pregnancy BMI, and PAL were analyzed as continuous variables. Education was categorized into two groups: ≥3 years of university studies and <3 years of university studies. Lactation referred to any type of lactation (exclusive or partial) and was categorized into three groups: 0–3.9, 4–8.9, and ≥9 months, respectively. Parity was categorized as nulliparous or parous. GWG was categorized into excessive, adequate, or insufficient, according to the guidelines of the Institute of Medicine (IOM) based on pre‐pregnancy BMI (IOM, 2009).

### 2.5 Statistical analysis

Descriptive statistics are presented as median and interquartile range (IQR, 25th and 75th percentiles). Changes in nutritional intake between third trimester of pregnancy and up to 18 months postpartum were analyzed by linear mixed models. Variables that were not normally distributed were log transformed before analysis. Each nutrient was set as a dependent variable and time points for study visit were included as repeated and fixed effects. Unstructured covariance for repeated effect was chosen.

Changes in nutritional quality from third trimester of pregnancy to 18 months postpartum were analyzed by linear mixed models where time points for study visit were set as repeated and fixed effect. Associations between selected determinants and changes in nutritional quality were analyzed by linear mixed models. Selected determinants were set as fixed effects and time points of study visits were set as repeated and fixed effects. Excessive GWG, parous, lactation ≥9 months and ≥3 years education at university were set as reference categories. For both changes in nutritional intake and quality, study visit in the third trimester of pregnancy was set as reference and Bonferroni adjustment for pairwise comparisons was used. The correlation and covariance matrix for nutritional quality at each study visit were assessed to determine the appropriate covariance structure. Bayesian information criterion (BIC) values were used to identify the best‐fitting covariance structure. The analysis indicated that the first‐order autoregressive structure provided the best fit to the data. To assess the model assumption of normally distributed residuals, quantile‐quantile plots were visually inspected.

The significance level was set to *p* < .05. All statistical analyses were executed using SPSS Statistics version 28.0 (IBM Corp., Armonk, NY, USA).

## 3 RESULTS

Study population, including reasons for dropout, missing data, and excluded cases is presented in Figure 1. The characteristics of the participants are presented in Table 1. Most of the participants (81%) had studied three or more years at university level. The majority (86%) had a normal pre‐pregnancy BMI. Only 13% were classified as overweight and none as obese. Median GWG was 12 (10–12) kg. Most participants lactated (exclusively or partially) for more than 4 months, with a median duration of 8 (7–11) months. At 2 weeks postpartum, 99% were lactating to some extent, and correspondingly 89% at 4 months, 17% at 12 months, and 1% at 18 months.

**TABLE 1:** Characteristics of the study population (*n* = 72).

	Median (p25–p75)
Age (years)^a^	32 (31–37)
Pre‐pregnancy body mass index (kg/m^2^)^b^	22 (21–24)
Physical activity level^c^	1.6 (1.5–1.7)

	*N* (%)
Education	
≤2 years of high school education	2 (3)
3 years of high school education	3 (4)
3 years of university education	9 (13)
≥3 years of university education	58 (81)
Pre‐pregnancy body mass index (kg/m^2^)^b^	
<18.5	1 (1)
18.5–24.9	62 (86)
≥25	9 (13)
Gestational weight gain^d^	
Insufficient	28 (39)
Adequate	24 (33)
Excessive	20 (28)
Nulliparity	36 (50)
Lactation (months)	
0–3.9	8 (11)
4–8.9	36 (50)
≥9	28 (39)
Smoking in third trimester of pregnancy	0 (0)

Abbreviation: P, percentile.

^a^ At 2 weeks postpartum.

^b^ Pre‐pregnancy weight was self‐reported.

^c^ In third trimester of pregnancy.

^d^ Calculated as weight in third trimester minus self‐reported pre‐pregnancy weight, categorized according to the guidelines of the Institute of Medicine, IOM (IOM, 2009).

### 3.1 Nutritional intake during pregnancy and postpartum

Overall, the E% from carbohydrates and fiber were higher in pregnancy compared to postpartum (Table 2). In contrast, the E% from protein and monounsaturated fat were lower in pregnancy compared to postpartum. Estimated intakes of vitamins A and C from foods were higher in pregnancy compared to most study visits postpartum, whereas for potassium estimated intakes were higher in pregnancy compared to 18 months postpartum.

**TABLE 2:** Nutritional intake and nutritional quality from foods during third trimester to 18 months postpartum, assessed by four‐day food diaries.

	3rd trimester (*n* = 72)	2 weeks pp (*n* = 72)	4 months pp (*n* = 70)	12 months pp (*n* = 63)	18 months pp (*n* = 50)
	Median (p25–p75)	Median (p25–p75)	Median (p25–p75)	Median (p25–p75)	Median (p25–p75)
Nutritional quality score^†^ ^,^ ^‡^	598 (531–651)	563 (464–636)	581 (486–640)	572 (477–642)	577 (531–613)
Energy (kcal)	2320 (2050–2620)^a,b^	2180 (1790–2520)^c^	2190 (1980–2440)^d^	2060 (1820–2520)^a^	2040 (1750–2300)^b,c,d^
Protein (E%)	15 (14–16)^a,b^	15 (14–16)^c,d^	16 (14–17)	16 (14–18)^a,c^	16 (15–18)^b,d^
Carbohydrate^§^ (E%)	47 (44–51)^a,b,c^	48 (45–52)^d,e,f^	45 (42–49)^a,d,g,h^	44 (40–48)^b,e,g^	42 (37–46)^c,f,h^
Fat^§^ (E%)	36 (32–38)^a^	34 (31–37)^b^	36 (32–40)	35 (32–40)	38 (34–41)^a,b^
Saturated fat^§^ (E%)	14 (12–15)	14 (12–16)^a^	15 (12–17)	15 (12–17)	15 (14–17)^a^
Monounsaturated fat^§^ (E%)	12 (10–13)^a,b^	12 (11–14)^c^	13 (11–14)^d^	13 (11–14)^a^	14 (12–15)^b,c,d^
Polyunsaturated fat^§^ (E%)	5 (4–6)^a,b^	3 (2–5)^a,c,d^	5 (4–6)^c^	5 (4–7)^d^	5 (4–7)^b^
Fiber (g/d)	22 (17–29)^a^	22 (16–28)^b^	20 (17–25)	20 (17–24)	19 (15–22)^a,b^
Added sugar (E%)	9 (7–13)^a^	10 (7–13)^b,c^	10 (7–14)^d^	9 (6–11)^b,e^	6 (5–10)^a,c,d,e^
Vitamin A^§^ (RE)	913 (685–1136)^a,b^	662 (480–844)^a,c^	825 (601–1200)^c,d^	751 (570–1001)	722 (547–842)^b,d^
Vitamin C (mg)	131 (101–190)^a,b,c,d^	92 (69–121)^a^	106 (66–138)^b,e^	82 (60–117)^c,e^	95 (55–129)^d^
Vitamin D^§^ (μg)	5.5 (3.9–7.4)	4.7 (3.0–6.4)^a^	5.6 (3.9–8.7)^a^	5.3 (3.4–7.7)	5.1 (3.8–7.7)
Vitamin E^§^ (α‐TE)	10 (8–12)	10 (8–13)^a^	12 (9–14)^a,b^	9 (8–12)^b^	10 (8–13)
Folate (μg)	280 (247–341)	258 (193–333)	267 (221–339)	259 (211–316)	264 (202–313)
Calcium^§^ (mg)	1029 (825–1282)	979 (742–1178)	1059 (804–1409)	980 (738–1205)	855 (675–1153)
Iron (mg)	11 (9–13)	11 (9–13)	10 (9–12)^a^	11 (10–14)^a^	10 (9–13)
Magnesium^§^ (mg)	331 (269–384)	336 (253–386)	336 (280–410)	332 (280–383)	310 (280–352)
Potassium (g)	3.3 (2.7–3.7)^a^	3.1 (2.5–3.6)	3.3 (2.7–3.8)	3.1 (2.7–3.6)	2.8 (2.5–3.3)^a^
Sodium (g)	2.8 (2.4–3.5)	2.8 (2.1–3.4)	2.8 (2.4–3.6)	2.9 (2.3–3.3)	2.7 (2.3–3.4)

*Note*: Statistical analyses were performed with linear mixed models and Bonferroni adjustment (*n* = 72). Within each variable, values followed by similar letters are significantly different (*p* < .05).

Abbreviations: E%, energy percentage; P, percentile; PP, postpartum.

^†^ According to NRF11.3 (Bianchi et al., 2020).

^‡^ Intakes adjusted to an energy intake of 2000 kcal.

^§^ Log transformed before linear mixed models' analysis.

The AR for vitamin A, C, E, and calcium were reached by >80% of the participants both in pregnancy and postpartum (Table 3). Over 60% reached the AR for folate and iron. However, less than one‐third reached the AR of vitamin D, and less than one‐fifth reached the RI for vitamin D, folate, and iron. Over time, 89%–96% of the participants exceeded the recommendation of maximum 10 E% from saturated fat, and only 15%–39% adhered to the RI for fiber (≥25 g/day). Thus, adherence to RIs of saturated fat, fiber, vitamin D, folate and iron were low during all study visits.

**TABLE 3:** Adherence to recommended intakes and average requirements of macro‐ and micronutrients from foods during third trimester to 18 months postpartum.

	3rd trimester (*n* = 72)		2 weeks pp (*n* = 72)		4 months pp (*n* = 70)		12 months pp (*n* = 63)		18 months pp (*n* = 50)	
	% RI^a^	% AR^b^	% RI^c^	% AR^b^	% RI^c^	% AR^b^	% RI^b^	% AR^b^	% RI^b^	% AR^b^
Protein	100	‐	97	‐	96	‐	92	‐	92	‐
Carbohydrate	74	‐	78	‐	54	‐	40	‐	32	‐
Fat	82	‐	89	‐	77	‐	73	‐	68	‐
Saturated fat	4	‐	11	‐	6	‐	6	‐	4	‐
Monounsaturated fat	79	‐	88	‐	84	‐	91	‐	96	‐
Polyunsaturated fat	42	‐	18	‐	54	‐	48	‐	58	‐
Fiber	35	‐	39	‐	23	‐	15	‐	22	‐
Added sugar	60	‐	49	‐	52	‐	66	‐	80	‐
Vitamin A	64	86	15	74	27	87	54	83	38	84
Vitamin C	82	90	43	89	53	91	60	81	68	78
Vitamin D	8	24	9	21	13	34	13	27	14	26
Vitamin E	47	100	43	100	56	99	78	100	76	100
Folate	0	87	0	68	2	80	3	79	4	78
Calcium	67	99	61	93	69	93	67	95	60	96
Iron	10^d^	75	15	67	7	63	21	80	6	63
Magnesium	74	‐	67	‐	76	‐	76	‐	72	‐
Potassium	58	‐	51	‐	60	‐	51	‐	44	‐
Sodium	24	‐	36	‐	28	‐	25	‐	36	‐

*Note*: Reference values (RI and AR) are from the Nordic nutrition recommendations 2012 (Nordic Council of Ministers, 2014).

Abbreviations: AR, average requirement; E%, energy percentage; PP, postpartum; RI, recommended intake.

^a^ During pregnancy.

^b^ For female individuals of reproductive age.

^c^ During lactation.

^d^ Pregnancy requires a minimum storage of 500 mg iron; supplementation may be indicated to meet the extra demands.

In the third trimester of pregnancy, 90% of the participants reported that they used some form of dietary supplement. The proportion was lower after delivery and ranged from 58% 2 weeks postpartum to 26% 18 months postpartum. Median intakes of vitamin D, iron, and calcium from supplements are presented in Table S1 and the total intake from foods and supplements are given in Table S2. Overall, most of the supplement users adhered to the ARs of vitamin D, calcium, and iron during pregnancy and postpartum. However, only 33% reported a total vitamin D intake above the RI at 18 months postpartum.

### 3.2 Determinants of nutritional quality changes from pregnancy to postpartum

No statistically significant changes in nutritional quality were found from the third trimester to the postpartum period (Table 2).

Age was positively related to nutritional quality changes over time (Table 4). In contrast, shorter duration of lactation was negatively related to nutritional quality over time when compared to those with a longer duration of lactation.

**TABLE 4:** Determinants of nutritional quality changes from third trimester of pregnancy to 18 months postpartum (*n* = 72).

	Nutritional quality changes by NRF11.3^a^		
	*β*	95% CI	*p*‐value^b^
Age (years)^c^	8	2, 14	0.006
Pre‐pregnancy body mass index (kg/m^2^)	6	−2, 14	0.155
Physical activity level^d^	33	−36, 103	0.349
IOM GWG groups^e^			
Insufficient	36	−11, 84	0.134
Adequate	33	−15, 81	0.174
Excessive	ref		
Education			
<3 years at university	9	−37, 55	0.701
≥3 years university	ref		
Parity			
Nulliparous	30	−9, 68	0.127
Parous	ref		
Lactation (months)			
0–3.9	−74	−140, −9	0.025
4–8.9	5	−32, 55	0.773
≥9	ref		

Abbreviations: CI, confidence interval; GWG, gestational weight gain; IOM, Institute of Medicine (IOM, 2009); NRF11.3, Nutrient Rich Food index 11.3 (Bianchi et al., 2020); ref, reference; β, β‐coefficient.

^a^ Intakes adjusted to an energy intake of 2000 kcal.

^b^ *p*‐value for linear mixed model analysis.

^c^ At two weeks postpartum.

^d^ At third trimester, 2 weeks, 4, 12 and 18 months postpartum.

^e^ Weight at study visit in trimester three minus self‐reported pre‐pregnancy weight.

## 4 DISCUSSION

This longitudinal study is the first to examine changes in nutritional intake and quality from pregnancy up to 18 months postpartum among individuals in Sweden. Nutritional intake changed from third trimester to the postpartum periods, whereas no changes in nutritional quality were found. Determinants of nutritional quality changes were age and lactation duration. Adherence to the RIs for saturated fat, fiber, vitamin D, folate and iron were low, both in late pregnancy and postpartum.

To our knowledge, this is the first study analyzing nutritional quality using the NRF11.3 index longitudinally during pregnancy and postpartum. A Japanese mother‐and‐child cohort recently used the NRF9.3 index to examine nutritional quality during mid‐pregnancy, including nine nutrients to encourage and three nutrients to limit (Imai et al., 2021). The authors found that fiber, iron, potassium, magnesium, and vitamin C were the major nutrients contributing to variation in the NRF9.3 index across the tertiles (Imai et al., 2021). We found decreased estimated intakes of fiber, vitamin A, vitamin C and potassium in the postpartum period compared to the third trimester. Since fruit and vegetables often contribute with a high content of these nutrients, our results may be consistent with the findings in the Japanese cohort (Imai et al., 2021) where food groups of fruit and vegetables were positively associated with the NRF index.

A Swedish study observed a decrease in fruit and vegetable intake from pregnancy to 6 months postpartum, accompanied by an increase in discretionary food consumption (Wennberg et al., 2016). The trend toward poorer overall dietary quality after delivery, aligns with our results and has been further corroborated by subsequent studies examining dietary quality during this transitional period (Lebrun et al., 2019; Poulain et al., 2021).

We found no significant changes in nutritional quality as a function of nutritional density during pregnancy and postpartum. Hence, the observed decreases in certain nutrient intakes postpartum might be explained by the decrease in energy intake. The immediate postpartum period is often marked by challenges, which are likely to influence food choices. A previous study by Poulain et al. demonstrated that the increased energy requirement of lactation was met by a higher energy intake, primarily derived from unhealthy food sources (Poulain et al., 2021). Our findings of a decrease in estimated energy intake postpartum may also be a consequence of motivation to return to pre‐pregnancy weight (Lim et al., 2015; Ohlendorf et al., 2012).

Short duration of lactation (<4 months) was negatively related to nutritional quality changes over time, compared with participants with longer duration of lactation. This result should be interpreted with caution since only 11% of the participants were categorized to the short duration of lactation category. However, our results are similar to other studies where exclusive breastfeeding, compared to exclusive formula feeding, was associated with postpartum dietary quality improvement (Wiltheiss et al., 2013). Previous studies have shown that individuals with higher education and normal pre‐pregnancy BMI are more likely to breastfeed for a longer period (Flacking et al., 2007; Reichental et al., 2021); characteristics that also are positively related to dietary quality during pregnancy (Doyle et al., 2017; Stråvik et al., 2019). Therefore, one cannot ascertain whether breastfeeding leads to higher nutritional quality or whether individuals with good compliance with nutritional recommendations also choose to breastfeed.

A higher pre‐pregnancy BMI is known to be associated with poor dietary quality during pregnancy (Englund‐Ogge et al., 2014; Northstone et al., 2008; Rifas‐Shiman et al., 2009; Tsigga et al., 2011). In our study, the median pre‐pregnancy BMI was 22 kg/m^2^, which is lower than the corresponding BMI for female individuals of child‐bearing age in the general Swedish population (25 kg/m^2^) (Statistics Sweden, 2020). Pre‐pregnancy BMI did not significantly determine nutritional quality changes in our model, possibly because the majority of participants had a normal pre‐pregnancy BMI. Due to small subgroups for the different BMI categories, sensitivity analyses regarding change in nutritional quality for participants with overweight or underweight were not possible.

Nutritional quality increased over time in older participants, which is consistent with earlier findings regarding healthy eating patterns during pregnancy (Arkkola et al., 2008; Bodnar & Siega‐Riz, 2002; Englund‐Ogge et al., 2014; Poulain et al., 2021; Rifas‐Shiman et al., 2009; Stråvik et al., 2019). Unlike results from previous research (Arkkola et al., 2008; Bodnar & Siega‐Riz, 2002; Englund‐Ogge et al., 2014; Northstone et al., 2008; Rifas‐Shiman et al., 2009; Stråvik et al., 2019), level of education did not significantly determine the changes in nutritional quality in our study. This is possibly explained by the overall high level of education, where 81% of the participants had ≥3 years of university education, compared to 37% from national data in the same age group (Statistics Sweden, 2021). Thus, the external validity of our study is limited by the fact that our study participants generally had a high education level and a higher proportion with a healthy pre‐pregnancy BMI than the general population.

The low median dietary intake of vitamin D throughout the study period is consistent with results from our more recent population‐based pregnancy cohort (Bärebring, Amberntsson, et al., 2018) and with data of female individuals of child‐bearing age in the national Swedish survey Riksmaten 2010–11 (Amcoff et al., 2012). Because of the low reported intakes of vitamin D in Sweden, an extension of the mandatory vitamin D fortification program was introduced by the National Food Agency in 2018 (The National Food Agency, 2020). The effect of the expanded vitamin D fortification is still unexplored. An additional source of vitamin D is through supplements. Among the supplement users in our study, over 70% had a total intake of vitamin D above the AR, whereas less than 35% had a vitamin D intake from food alone that reached the AR. This indicates that vitamin D supplements contributed to the total vitamin D intake. In addition to dietary and supplement sources, vitamin D can be synthesized in human skin upon exposure to solar ultraviolet‐B radiation (Prentice et al., 2008). While our study did not address the contribution of sun exposure, it should be taken into consideration that insufficient vitamin D intake not necessarily lead to inadequate vitamin D status (Prentice et al., 2008).

In our study, nearly 70% reported a folate intake above the AR at all study visits, whereas only 0–4% reached the RI in pregnancy and postpartum. This is in accordance with Riksmaten, the national Swedish survey, where only 3% of female individuals in reproductive age reached the RI of folate (Öhrvik et al., 2018). The low median dietary folate intake in our study is also consistent with previous findings among pregnant individuals (Lundqvist et al., 2014), where median folate intake from food was 277 μg/day. In Sweden, folic acid supplementation is recommended from preconception until gestational week 12 (The National Food Agency, 2008). However, both our results and those of others, highlight the challenge of reaching the RI for folate solely through food during pregnancy and lactation, as well as among female individuals of reproductive age (Augustin et al., 2020; Lundqvist et al., 2014; Öhrvik et al., 2018; Stråvik et al., 2019).

Dietary supplements were not included in the NRF11.3 analyses, since the intention of NRF is to examine the nutritional quality from foods (Fulgoni 3rd et al., 2009). Although the overall dietary quality should primarily be improved, dietary supplements may be warranted for some individuals as both we, and others (Augustin et al., 2020; Stråvik et al., 2019) have shown that the intake of certain nutrients during pregnancy and postpartum are lower than recommended.

### 4.1 Strengths and limitations

The major strengths of this study are the longitudinal design with repeated assessments of dietary intake during a long follow‐up period from pregnancy to 18 months postpartum. The detailed dietary intake data from the four‐day food diaries made it possible to present actual intakes instead of food habits. In addition, the prospective method of a food diary minimizes the risk for recall biases (Gibson, 2005). Another strength was the use of linear mixed models which includes the full data set to diminish risk in undermining statistical power. Lastly, this is the first study to our knowledge that examines overall nutritional quality through the NRF11.3 index among pregnant individuals in Sweden, and with the 18 months follow‐up postpartum.

Limitations include the small sample size and a homogeneous highly educated study population with healthy pre‐pregnancy BMI, and therefore the generalizability of our results may be limited. Nevertheless, we found that less than 20% of participants adhered to the RIs for saturated fat, vitamin D, folate, and iron. Thus, there is a need for increasing the public awareness of the importance of these nutrients. Another limitation is that the estimations of nutritional intake are based on self‐reported food diaries where risks of misreporting and social desirability bias cannot be ignored (Gibson, 2005). Participants in this study were their own control, which could possibly reduce the individual differences in misreporting. Also, assessment of nutritional status through biochemical indicators to infer potential deficiencies would have complemented the self‐reported dietary intake data, as a low reported nutrient intake does not necessarily equate to a poor nutritional status (Lynch et al., 2018; Prentice et al., 2008). It is conceivable that the motivation for healthy lifestyle changes increases during pregnancy (Lindqvist et al., 2017). Thus, it would have been preferable to use pre‐pregnancy dietary intake as reference category. Unfortunately, no pre‐pregnancy data were collected in this study. However, only small changes in dietary quality have previously been observed between pre‐pregnancy and during pregnancy (Crozier et al., 2009). A general challenge of evaluating dietary intake and quality during the cycle of reproduction is that nutrient requirements change from pregnancy to postpartum. Therefore, to study the longitudinal changes in nutritional quality, rather than the changes in nutrient requirements, the reference values for NRF11.3 were kept constant for all study visits. This means that an overestimation in the score of NRF11.3 may have occurred since the RI for some nutrients is higher during pregnancy and lactation, compared to post weaning. We selected reference values for female individuals of reproductive age due to the limited presence of lactation at 12 and 18 months postpartum. If reference values for pregnancy or lactation had been used, we suppose that the NRF11.3 score could have been underestimated. Lastly, due to difficulties in estimations of sodium intake, the score of NRF11.3 may be somewhat overestimated. However, we do not believe that this would alter the interpretation of the longitudinal changes in nutritional quality.

## 5 CONCLUSIONS

In conclusion, the nutritional intake changed from late pregnancy to 18 months postpartum among Swedish individuals. However, the nutritional quality remained relatively stable over time. Age and lactation duration were the main determinants of changes in nutritional quality. Less than one‐fifth of the participants adhered to the RIs of saturated fat, vitamin D, folate, and iron. Future studies should include a larger and more diverse study population and focus on the identification of people at risk of adverse dietary changes from pregnancy to the postpartum period.

## AUTHOR CONTRIBUTIONS

**Mathilda Forsby:** Data curation (supporting); formal analysis (lead); methodology (supporting); visualization (lead); writing – original draft (lead); writing – review and editing (lead). **Erik Hulander:** Formal analysis (supporting); methodology (supporting); visualization (supporting); writing – original draft (supporting); writing – review and editing (supporting). **Anna Amberntsson:** Data curation (supporting); formal analysis (supporting); methodology (supporting); visualization (supporting); writing – original draft (supporting); writing – review and editing (supporting). **Petra Brembeck:** Data curation (lead); investigation (supporting); methodology (supporting); project administration (supporting); resources (supporting); supervision (supporting); visualization (supporting); writing – original draft (supporting); writing – review and editing (supporting). **Anna Winkvist:** Conceptualization (supporting); funding acquisition (supporting); investigation (supporting); methodology (supporting); project administration (supporting); resources (supporting); supervision (supporting); visualization (supporting); writing – original draft (supporting); writing – review and editing (supporting). **Linnea Bärebring:** Data curation (supporting); formal analysis (supporting); methodology (supporting); supervision (supporting); visualization (supporting); writing – original draft (supporting); writing – review and editing (supporting). **Hanna Augustin:** Conceptualization (lead); data curation (lead); formal analysis (supporting); funding acquisition (lead); investigation (lead); methodology (lead); project administration (lead); resources (lead); supervision (lead); visualization (supporting); writing – original draft (supporting); writing – review and editing (supporting).

## FUNDING INFORMATION

This research was funded by the Graduate School Environment and Health, Martina and Wilhelm Lundgrens Foundation, Hvitfeldtska Foundation, Magnus Bergvall Foundation, Fredrik and Ingrid Thuring Foundation and The Swedish Research Council for Sustainable Development Formas.

## CONFLICT OF INTEREST STATEMENT

None declared.

## Supporting information


Appendix S1.

## Data Availability

The data that support the findings of this study are available from the corresponding author upon reasonable request.
