# Supplementary material for: Nutritional intake and determinants of nutritional quality changes from pregnancy to postpartum—a longitudinal study
Source: Food Sci Nutr. 2023 Nov 20;12(2):1245–56. doi: 10.1002/fsn3.3838 (PMC10867539; doi:10.1002/fsn3.3838)
Supplement: Supplementary file 1 — Appendix S1. [file FSN3-12-1245-s001.docx]

**Supporting information for Nutritional intake and determinants of nutritional quality changes from pregnancy to postpartum - a longitudinal study**

**NRF11.3 = NR11 – LIM3**

$$\boldsymbol{NR}\boldsymbol{11:} \sum_{i=1-11}=\left( {Nutrient}_{i} /{DRI}_{i} \right)*100$$

NR11: protein, fiber, vitamin A, C, D, E, folate, calcium, iron, magnesium, and potassium

*Nutrient_i_ =* reported intake of nutrient *i*

*DRI_i_* = dietary reference intake for nutrient *i*

$$\boldsymbol{LIM}\boldsymbol{3:} \sum_{i=1-3}=\left( {Nutrient}_{i} /{MRI}_{i} \right)*100$$

LIM3: saturated fat, added sugar and sodium

*Nutrient_i_ =* reported intake of nutrient *i*

*MRI_i_* = dietary reference intake for nutrient *i*

**Appendix figure 1.** The algorithm for calculating the nutrient rich food index 11.3 (NRF11.3) (Bianchi et al., 2020). Abbreviations: NR, nutrient rich; DRI, dietary reference intake; LIM, limiting nutrients; MRI, maximum recommended intake.

**Appendix table 1.** Reported use of supplements and median (p25-p75) intake from supplements during third trimester of pregnancy to 18 months postpartum (n=72).

|  | **3^rd^ trimester** | | **2 weeks pp** | | **4 months pp** | | **12 months pp** | | **18 months pp** | |
| --- | --- | --- | --- | --- | --- | --- | --- | --- | --- | --- |
|  | **N (%)** | **Median**  **(p25-p75)** | **N (%)** | **Median**  **(p25-p75)** | **N (%)** | **Median**  **(p25-p75)** | **N (%)** | **Median**  **(p25-p75)** | **N (%)** | **Median**  **(p25-p75)** |
| Vitamin D (µg) | 39 (54) | 5 (4-8) | 29 (40) | 5 (4-8) | 21 (29) | 4 (2-8) | 16 (24) | 4 (3-6) | 9 (17) | 3 (1-6) |
| Iron (mg) | 58 (81) | 100 (26-115) | 35 (49) | 18 (8-100) | 23 (32) | 8 (5-18) | 12 (18) | 8 (8-17) | 9 (17) | 6 (2-10) |
| Calcium (mg) | 36 (50) | 200 (120-240) | 29 (40) | 126 (120-200) | 21 (30) | 120 (68-245) | 13 (19) | 120 (116-238) | 11 (20) | 120 (51-214) |

Abbreviations: PP, postpartum; P, percentile.

**Appendix table 2.** Reported median (p25-p75) intake, adherence to the recommended intakes, and the average requirements from foods and supplements during third trimester of pregnancy to 18 months postpartum among supplement users.

|  | **3^rd^ trimester** | | | **2 weeks pp** | | | **4 months pp** | | | **12 months pp** | | | **18 months pp** | | |
| --- | --- | --- | --- | --- | --- | --- | --- | --- | --- | --- | --- | --- | --- | --- | --- |
|  | **Median (p25-p75)** | **% RI^†^** | **% AR^‡^** | **Median (p25-p75)** | **% RI^§^** | **% AR^‡^** | **Median (p25-p75)** | **% RI^§^** | **% AR^‡^** | **Median (p25-p75)** | **% RI^‡^** | **% AR^‡^** | **Median (p25-p75)** | **% RI^‡^** | **% AR^‡^** |
| Vitamin D (µg) | 13 (9-14) | 69 | 87 | 10 (8-12) | 55 | 79 | 12 (8-15) | 70 | 80 | 11 (6-13) | 53 | 73 | 9 (6-13) | 33 | 78 |
| Iron (mg) | 112 (32-124) | 97 | 100 | 27 (18-113) | 89 | 100 | 21 (13-27) | 68 | 96 | 20 (17-29) | 92 | 100 | 17 (13-23) | 57 | 100 |
| Calcium (mg) | 1193 (990-1614) | 83 | 97 | 1267 (1040-1438) | 83 | 97 | 1318 (981-1759) | 85 | 100 | 1182 (798-1351) | 77 | 100 | 1197 (973- 1504) | 82 | 100 |

Abbreviations: PP, postpartum; RI, recommended intake; AR, average requirement. Reference values (RI and AR) from the Nordic Nutrition recommendations 2012 (Nordic Council of Ministers, 2014).

^†^During pregnancy

‡For female individuals of reproductive age;

§During lactation;

¶Pregnancy requires a minimum storage of 500 mg iron; supplementation may be indicated to meet the extra demands;
